# Intracranial Meningioma Diagnosed during Pregnancy Caused Maternal Death

**DOI:** 10.1155/2014/158326

**Published:** 2014-09-10

**Authors:** Zehra Kurdoglu, Orkun Cetin, Ismail Gulsen, Deniz Dirik, M. Deniz Bulut

**Affiliations:** ^1^Department of Obstetrics and Gynecology, Faculty of Medicine, Yuzuncu Yil University, 65080 Van, Turkey; ^2^Yuzuncu Yil Universitesi Tip Fakultesi, Kadin Hastaliklari ve Dogum Anabilim Dali, Dursun Odabas Tip Merkezi, Kampus, 65080 Van, Turkey; ^3^Department of Neurosurgery, Faculty of Medicine, Yuzuncu Yil University, 65080 Van, Turkey; ^4^Department of Radiology, Faculty of Medicine, Yuzuncu Yil University, 65080 Van, Turkey

## Abstract

Brain tumors are rarely diagnosed during pregnancy. Accelerated growth of intracranial meningiomas during pregnancy sometimes requires urgent surgical intervention. We describe a 41-year-old pregnant woman with severe neurological decompensation requiring immediate neurosurgery. Cesarean section resulted in maternal death. Meningioma diagnosed during a viable pregnancy should be managed according to the severity of maternal neurological symptoms and gestational age of pregnancy. Early intervention for intracranial tumors during pregnancy may save maternal and fetal lives.

## 1. Introduction

Intracranial tumors during pregnancy were first described in 1898 [1]. Although primary intracranial neoplasms are seen rarely, they are the fifth leading cause of cancer-related death in women aged 20 to 39 years [2]. Glial neoplasms are the most common tumors, followed by meningiomas and acoustic neuromas [3].

Meningiomas may exhibit accelerated growth during the second half of pregnancy [4, 5]. Neurosurgery has become feasible through advanced anesthetic techniques for meningiomas during pregnancy. Urgent neurosurgery is reserved for signs of maternal progressive neurologic deficit [6].

In the present paper, we report a 41-year-old, 39-week pregnant woman with intracranial meningioma and progressive severe neurologic signs leading to maternal death during pregnancy.

## 2. Case

**Figure 1:**
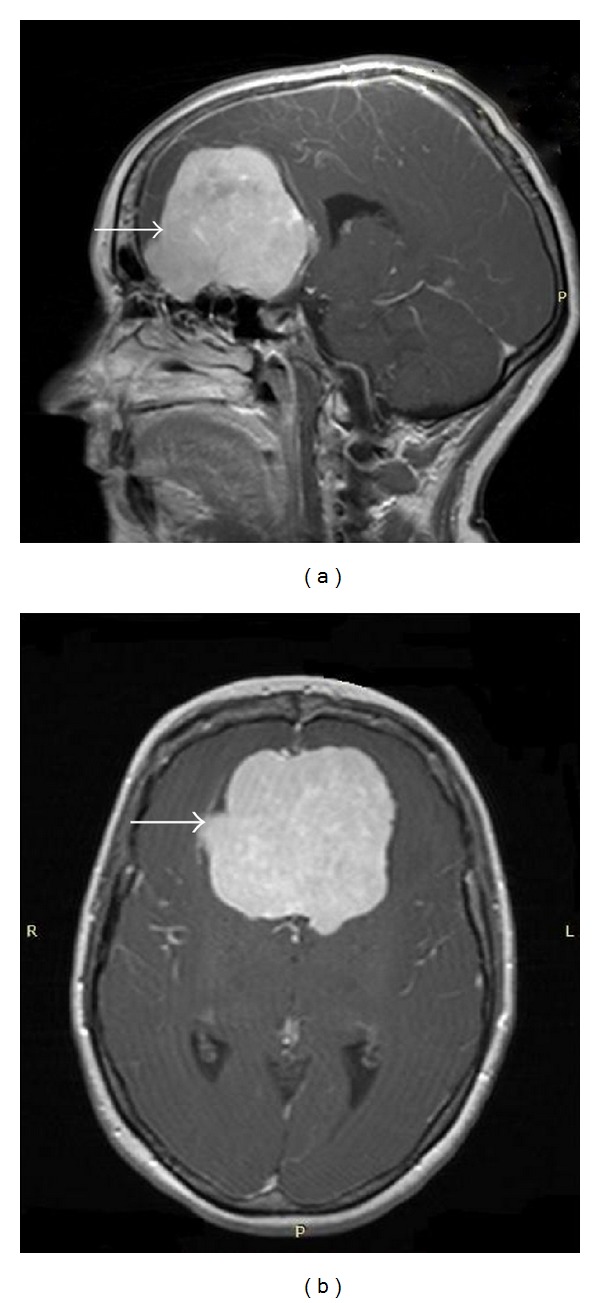
Arrows show a large lobulated extra-axial tumor compressing the olfactory sulcus in gadolinium-enhanced T1-weighted MRI.

A 41-year-old woman, gravida 5, parity 4, at 39 weeks of gestation presented with progressive visual impairment and confusion. On the neurological examination, there were focal deficits and symptoms related to raised intracranial pressure. Magnetic resonance imaging (MRI) with gadolinium showed a mass lesion with compression of olfactory sulcus, consistent with meningioma (Figures 1(a) and 1(b)). Because of her progressive visual impairment and confusion, both emergent neurosurgery and cesarean section were planned at the same time. A 3100 g and 7–9 Apgar score healthy infant was delivered by cesarean section firstly. Then craniotomy was performed for excision of the tumor. The pathology report described meningothelial meningioma, World Health Organization (WHO) grade 1. On the third day after the operation, persistent hypotension was observed following recurrent epileptic seizures in the Intensive Care Unit. The patient did not respond to aggressive medical treatment of the hypotension. Hypoperfusion of the central nervous system led to diffuse brain infarction and maternal death.

## 3. Discussion

Growth of meningiomas accelerates during pregnancy [7]. The mechanisms of tumor growth remain controversial. Authors have concluded that reversible hemodynamic changes such as hypervascularity and intracellular and/or extracellular edema were most likely responsible for the rapid growth pattern. A progesterone-induced mechanism has also been reported to be responsible for enlargement of meningiomas in pregnancy [8, 9]. After delivery, regression of tumor size and resolution of symptoms have been described [10].

The clinical picture of headache, vomiting, or seizures can be confused with hyperemesis gravidarum in early pregnancy or preeclampsia in late pregnancy. However, the presence of abnormal findings on fundoscopic examinations, visual impairment, limb weakness, focal seizures, and neurological deficits should alert physicians to the possibility of an intracranial lesion and prompt further investigation with MRI for diagnosis.

Most investigators share the same general management paradigm for intracranial tumors manifested and diagnosed during pregnancy [11]. There is agreement that if acute and severe maternal neurological deterioration develop, a life-saving procedure is mandatory, regardless of any other factors. In these rare cases, two factors should be emphasized for the timing for neurosurgery: maternal neurological conditions and gestational age of the fetus. In our case, although neurosurgery and cesarean section had been planned immediately due to progressive neurologic decompensation signs, maternal death occurred.

In conclusion, meningioma diagnosed during a viable pregnancy should be managed according to the severity of maternal neurologic symptoms and gestational age of pregnancy. Early intervention for intracranial tumors during pregnancy may save maternal and fetal lives. A multidisciplinary approach involving a neurosurgeon, an obstetrician, and an anesthesiologist is required to improve maternal and fetal well-being.
